# Transcriptome Profiling Analyses in Psoriasis: A Dynamic Contribution of Keratinocytes to the Pathogenesis

**DOI:** 10.3390/genes11101155

**Published:** 2020-09-30

**Authors:** Geneviève Rioux, Zainab Ridha, Mélissa Simard, Florence Turgeon, Sylvain L. Guérin, Roxane Pouliot

**Affiliations:** 1 Centre de Recherche en Organogénèse Expérimentale de l’Université Laval/LOEX, Axe Médecine Régénératrice, Centre de Recherche du CHU de Québec, Québec, QC G1J 1Z4, Canada; genevieve.rioux.9@ulaval.ca (G.R.); zainab.ridha.1@ulaval.ca (Z.R.); melissa.simard.6@ulaval.ca (M.S.); florence.turgeon.1@ulaval.ca (F.T.); sylvain.guerin@fmed.ulaval.ca (S.L.G.); 2 Faculté de Pharmacie, Université Laval, Québec, QC G1V 0A6, Canada; 3 Centre Universitaire d’Ophtalmologie-Recherche, Centre de Recherche du CHU de Québec, Axe Médecine Régénératrice, Québec, QC G1S 4L8, Canada; 4 Faculté de Médecine, Département d’Ophtalmologie, Université Laval, Québec, QC G1V 0A6, Canada

**Keywords:** psoriasis, transcriptome, keratinocytes, skin, gene expression, microarray, RNA-seq

## Abstract

Psoriasis is an immune-mediated inflammatory skin disease with a complex etiology involving environmental and genetic factors. A better insight into related genomic alteration helps design precise therapies leading to better treatment outcome. Gene expression in psoriasis can provide relevant information about the altered expression of mRNA transcripts, thus giving new insights into the disease onset. Techniques for transcriptome analyses, such as microarray and RNA sequencing (RNA-seq), are relevant tools for the discovery of new biomarkers as well as new therapeutic targets. This review summarizes the findings related to the contribution of keratinocytes in the pathogenesis of psoriasis by an in-depth review of studies that have examined psoriatic transcriptomes in the past years. It also provides valuable information on reconstructed 3D psoriatic skin models using cells isolated from psoriatic patients for transcriptomic studies.

## 1. Introduction

Psoriasis is a common immune-mediated inflammatory skin disease characterized by well-demarcated scaly raised plaques [1]. The disease has a different prevalence, depending on ethnicity and geographical distribution. Plaque, erythrodermic, pustular, inverse and guttate psoriasis are the diverse subtypes of psoriasis, according to the onset of the disease. Plaque psoriasis, also known as psoriasis vulgaris, is the most common clinical subtype, affecting about 90% of patients [2,3]. The lesions are well-defined papulosquamous scales characterized by the focal formation of erythematous, raised plaques that constantly shed scales derived from excessive growth of skin epithelial cells [4]. The growth and dilation of superficial blood vessels, which causes redness, and the hyperplasia of the epidermis explain the clinical features of this chronic disease. The most affected body parts include elbows, knees, scalp, sacroiliac, lower back and nails [5]. In psoriasis vulgaris, the plaques are generally symmetrically distributed, suggesting a connection with the nervous system. The skin lesions develop after the immune system sends incorrect signals, increasing the mitotic rate of keratinocytes, leading to incomplete cornification and to a poorly adherent stratum corneum [6]. Psoriasis impacts general health since it is related to many other comorbidities, such as psoriatic arthritis, inflammatory bowel disease, hypertension, atherogenic dyslipidemia, diabetes, and many others [7]. Affected patients can also suffer from depression and anxiety. Therefore, psychological consequences have to be considered [8]. This skin disease has been recognized as a T lymphocyte mediated autoimmune skin condition. It is caused by various cutaneous cellular changes, including epidermal keratinocyte hyperplasia, T lymphocyte infiltration, vascular hyperplasia, presence of neutrophils, and other forms of leucocytes in infected skin [4]. The exact etiology is not yet fully understood [9]. However, it is conceivable to recognize specific components involved in the development of this polygenic condition, such as genetic and environmental factors along with immune system defects [6,10]. Abnormal epidermal hyperplasia and dysfunctional immune response leading to cell infiltration in the dermis and epidermis are two examples resulting from this altered crosstalk between keratinocytes and immune cells [6].

Although psoriasis is an incurable disease, better insight on related genomic alterations help design precise therapies to accomplish better treatment outcomes. Gene expression can provide relevant information about the altered expression of mRNA transcripts, thus giving new insights into the disease onset. Transcriptome analysis techniques, such as microarrays and RNA-sequencing (RNA-seq), serve as relevant tools for the discovery of new biomarkers as well as the discovery of new therapeutic targets. This review focuses on discoveries related to the contribution of keratinocytes in the molecular pathogenesis of psoriasis by an in-depth review of studies that have examined the psoriasis transcriptome between 2001 and 2020.

## 2. The Genetic Basis of Psoriasis

### 2.1. Genetic Factors

The worldwide prevalence of psoriasis is about 2%. More than 125 million individuals are affected, with an estimated heritability between 66% and 90% [11,12,13]. Epidemiological studies involving cohorts of 95 or more subjects have found a higher concordance rate among monozygotic twins compared to dizygotic twins (35–72% vs. 15–23%, respectively) [14,15,16]. Moreover, a significantly higher incidence of the disease is observed among patients’ relatives [17]. The assessment of the sera of 346 subjects from a kindred of 815 Caucasian Americans spanning six generations has highlighted the implication of genetic factors [18]. Although psoriasis can affect people of all ages, there is a bimodal age of onset: early-onset occurs before 40 years old and represents 75% of the cases, while the late-onset occurs at a mean age of 56–60 years [19,20]. The role of molecular genetics in psoriasis has been proven to be involved in the disease’s pathology in previous years. Large-scale studies involving more than 2600 and 5000 psoriatic individuals have identified multiple loci associated with psoriasis in the human genome [21,22]. The principal locus that has been demonstrated to be connected to the disease is PSORS1, encoding the gene variant *HLA-Cw6*, which is carried by up to 85% of patients with early-onset psoriasis in comparison with 15% in late-onset psoriasis [19]. More recently, further studies consisting of over 15,000 psoriatic cases carried out by Tsoi and his colleagues have led to the identification of additional loci associated with psoriasis, raising the number of documented psoriasis susceptibility loci to 41 in Caucasians and 49 worldwide [23,24].

### 2.2. Differences between Men and Women

The prevalence of psoriasis is considered to be balanced between men and women [25]. Only a few studies in the literature explored the differences in genetic risks between the two sexes. However, some have pointed out sex-related differences in the severity of psoriasis and divergent genetic factors. In a study involving 369 patients with familial psoriasis, Gudjonsson et al. stated that *HLA-Cw6*-positive female patients may have an earlier onset of psoriasis than *HLA-Cw6*-positive males [26]. Research by Quiero et al. on psoriasis arthritis (PsA) showed variations in gender distribution of certain genetic markers from the major histocompatibility complex (MHC) region when patients were separated by age at the beginning of the disorder [27]. Moreover, Huffmeier et al. found a significantly higher concentration of the PTPN22*620W allele carriers in males than in females in a study of 375 patients with PsA [28]. Furthermore, the higher proportion of men with access to systemic treatment compared to women has raised questions about the severity of psoriasis between both genders. Studies have confirmed that psoriasis affects men more severely than women by highlighting a superior median of the psoriasis area severity index (PASI) score in men than in women [29,30,31]. A study investigating 461 psoriatic patients revealed a strong association between the score value rs1062470AA genotype (*PSORS1C1*/*CDSN*) and the PASI score in males only, increasing their risk of disease severity and pointing out that it might be gender-dependent [30]. Males with AA genotype had a significantly higher PASI score in comparison to others. This was not observed in female patients. Interestingly, independently of rs1062470 genotype, males had considerably higher PASI scores than females except for the earliest onset of the disease, suggesting that psoriasis is more severe in men than in women [31,32]. Another association of rs887466 (*PSORS1C3*) with psoriasis risk in men and women was noted by Wiśniewski and his colleagues [30]. The protective effect of the A allele and AA genotype was only visible for men. However, it remains unclear why this effect is only observed in male patients. Other studies, each involving around 50 male patients with psoriasis, have linked serum testosterone levels to psoriasis, suggesting the involvement of sex hormones in the pathology [33,34]. Finally, in a study of 121 male patients with psoriasis, Allam et al. also reported that the severity of psoriasis is inversely correlated with serum testosterone levels in men [35].

### 2.3. Genes Related to Immune Response

Psoriasis undoubtedly has an immunological component since therapies targeting T cell activation as well as effector cytokines produced by these cells were shown to be efficient in this disease [36,37]. Genome-wide association studies have also revealed that several genes involved in the pathology were linked to the immune system [21,22]. IL-23 is an upstream regulatory cytokine that promotes the survival and expansion of IL-17-producing T cells. Three genes involved in IL-23 signaling, namely *IL12B* (encoding the p40 subunit common to IL-12 and IL-23), *IL23A* (encoding the p19 subunit of IL-23) and *IL23R* (encoding a subunit of the IL-23 receptor), have a confirmed association with the disease [22]. Moreover, single-nucleotide polymorphisms (SNPs) located in the 3’-untranslated-region as well as ~60kb upstream of the *IL12B* coding region mark risk haplotypes (rs3212227 and rs6887695, respectively), while R381Q amino acid substitution within the *IL23R* gene provides a protective role against psoriasis [38,39]. Furthermore, the role of innate immunity in the pathogenesis of psoriasis has been put forward by Tsoi and his colleagues with the identification of additional psoriasis susceptibility loci [24]. Among the newly identified loci, five are specifically associated with psoriasis and are involved in innate immune responses (*DDX58*, *KLF4*, *ZC3H12C*, *CARD14* and *CARM1*). In addition, three genes that are part of an immunoregulatory network downstream of the IL-17 receptor have been identified as potential psoriasis candidate genes (*TRAF3IP2*, *NFKBIZ* and *TNFAIP3*). Finally, the abundant production of TNFα in psoriatic skin is a key feature of the disease. Studies have revealed an association between psoriasis and polymorphisms in the TNFα promoter region, affecting its production and therefore suggesting a disturbance in the recognition of regulatory DNA target sites by transcription factors important for the expression of that gene. More specifically, the replacement of guanine with adenine in position-238 is linked to a higher production of TNFα, and consequently to a higher risk of psoriasis in the Caucasian population [40,41]. However, as reported by Liu et al., the gene that encodes TNFα lies in the MHC region and has an association with human leukocyte antigen (HLA) class I alleles such as HLA-C. Therefore, these associations could be due to the proximity with HLA-C [42].

## 3. Implication of Keratinocytes

Over the years, keratinocytes were discovered to be crucial in the initiation, maintenance, and regulation of immune skin reactions. They act as an executor in response to inflammatory mediators, including IL-17 and IL-36, for developing the full-blown psoriatic phenotype. Keratinocytes also produce a variety of cytokines, chemokines and antimicrobial peptides that participate in the amplification of the local inflammatory response and the maintenance of an inflammatory cascade by maintaining epidermal hyperplasia [43,44]. The explanation of psoriasis is constantly fluctuating between keratinocytes and immune cells. Before 1970, keratinocytes were commonly acknowledged as the main actor in the evolution of psoriasis. However, T cells were later discovered to play a role in the pathogenesis. Therefore, it is now believed that the crosstalk between keratinocytes and immune cells, particularly T cells and dendritic cells, plays critical roles in the pathogenesis of psoriasis [45]. Anne Bowcock, a geneticist at Washington University in St. Louis, gave the first indication that keratinocytes play a major role in the development of psoriasis. Indeed, she revealed that two mutations in the gene encoding for the caspase recruitment domain-containing protein 14 (*CARD14*), are primarily observed in keratinocytes and might induce psoriasis by triggering expression of NF-κB, an inducible transcription factor that regulates a large array of genes involved in different processes of the immune and inflammatory responses [46,47]. About twenty *CARD14* variants were found in patients suffering from psoriasis [48]. Some mutations constitutively activated CARD14 by self-aggregation, resulting in the expression of pro-inflammatory factors and the development of psoriasis [48,49]. Furthermore, a mutation of glutamic acid in position 138 in the coiled-coil domain of CARD14 *(Card14ΔE138*) results in a gain-of-function mutation. This mutation leads to hyperactivation of CARD14 and is sufficient to orchestrate the complex processes that drive IL-23/IL-17-mediated psoriasiform skin inflammation in vivo [50]. These results help identify a variety of genes involved in innate immunity, emphasizing the importance of keratinocytes as potential initiators of psoriasis. Moreover, Gilliet and his colleagues established that keratinocytes play a role in the initiation of psoriasis after injury by secreting the antimicrobial peptides LL-37 conjugated with self-DNA to activate toll-like receptor 9 in plasmacytoid dendritic cells [51]. Cutaneous injury activates toll-like receptor 3 in keratinocytes to produce various proinflammatory cytokines, such as TNF-α, IL-6, and IL-36. The production of psoriasis-associated inflammatory cytokines can also be induced by mechanical stretches [52,53]. These studies confirm that cytokines produced by keratinocytes can play a significant role in the development of this condition, and that keratinocytes are important in the initiation of cutaneous inflammation by activating immune cells.

Experiments on animal models have also contributed to diminishing the importance attributed to immune cells as key instigators of skin inflammation in psoriasis. Indeed, studies in mice shortly after birth revealed that the specific ablation of IκB kinase (IKK)2 in keratinocytes (*K14-Cre/Ikk2FL/FL*) leads to the development of plaques similar to psoriasis on the dorsal skin of mice. The deletion of IKK2 in keratinocytes leads to an uncontrolled TNFR1 signal in them, producing a large amount of IL-24 [54,55]. Likewise, studies by Wagner and his colleagues revealed that epidermal deletion of the AP1 transcription factor subunits JunB and c-Jun lead to a phenotype resembling the histologic and molecular hallmarks of psoriasis in adult mice. Rag2-deficient JunB/c-Jun double-mutant mice showed epidermal thickening, altered keratinocyte differentiation, vascular dilatation and slight epidermal abscesses [56]. The debate over whether abnormalities in the keratinocytes or of the immune system are responsible for triggering the disease is still emerging, suggesting a dynamic contribution of keratinocytes to the pathogenesis [45,46,57,58].

## 4. Transcriptome Profiling Analysis for a Better Understanding of the Pathogenesis

### 4.1. Overview of Transcriptomic Studies

Transcriptome profiling is a widely used technique for generating hypotheses on molecular mechanisms associated with psoriasis. The common purpose of transcriptomic studies is to compare gene expression profiles between disorders to detect differentially expressed genes (DEGs). The first transcriptomic study providing a list of deregulated genes in psoriasis was performed by Oestreicher and his colleagues [59]. Subsequently, further studies followed, highlighting the altered expression of several genes as well as the signaling pathways affected in the pathology of psoriasis (Table 1). The interesting data resulting from these transcriptomic studies allowed for a better understanding of the mechanisms involved in the disease. Moreover, transcriptomics serves as a relevant tool for the discovery of potential therapeutic targets. Previously published transcriptomic studies are presented and discussed in Table 1.

Recently, additional expertise related to the pathomechanisms of psoriasis has arisen from research on functional genomics including the profiling of RNA expression. Early knowledge of the molecular contributors of this pathology came from data generated by microarray experiments. Various microarrays comprising different oligonucleotide probe sets have been widely used to study skin biopsies’ transcriptome (Table 1). In the early 2010s, studies have slowly progressed towards the use of high-throughput complementary DNA sequencing (RNA-seq). The first studies that applied RNA-seq to better understand the molecular basis of psoriasis identified altered expression of a variety of new miRNAs and genes in psoriasis, suggesting a promising application to reveal the contribution of novel transcripts [69,71]. In this sense, Li et al. have undertaken a large-scale study to compare the data from RNA-seq versus those from microarrays. According to their study, results obtained with RNA-seq are consistent for the intermediate- and high-abundance transcripts with those of microarrays. However, RNA-seq offers higher sensitivity by revealing several transcripts with low expression levels that were not identified by microarray analysis [72]. In recent years, single-cell RNA-seq has revolutionized transcriptomic studies. This new technology offers the unique opportunity to dissect the dynamic gene expression at single-cell resolution. Indeed, the skin is a particularly complex multicellular system made up of various cell subpopulations with different states. The expression profile of these different cells is affected, among other things, by cells at different stages of the cell cycle, divergent response to stimuli, as well as temporal and spatial organization allowing communication and interaction with each other [82]. Using single-cell RNA-seq, an interesting study by Cheng et al. compared the epidermis from different anatomical sites and the psoriatic epidermis [77]. Regarding human skin, information remains incomplete concerning the heterogeneity of the different epidermal populations, their hierarchical relationship as well as the existence of rare populations and their localization in the epidermis [83]. The results emerging from this study provide valuable information on the state of human epidermal cells and their differentiation.

### 4.2. Studying the Contribution of Keratinocytes in Psoriasis

Since 2001, various research teams have been comparing healthy and psoriatic skin transcriptomes to investigate the molecular mechanisms of the pathology. Ostreicher et al. were the first to undertake large-scale gene expression profile analysis in the context of psoriasis [59]. They drew up a comprehensive list of 159 genes whose expression was altered, and which defined the pathology from a molecular perspective. To facilitate the understanding of the involved mechanisms, the authors also presented the results using a color code to distinguish the frequency levels of the listed genes. The color code sheds light on the heterogeneous nature of cell populations present in skin biopsies by making it possible to discern genes originating from rare cell populations since it can impact the observed gene expression variations. Indeed, a slight fold change of low-frequency genes can lead to major changes in rare cell populations. Using this method, Ostreicher et al. were able to establish a list of differentially expressed genes between lesional and non-lesional psoriatic skin, some of which are newly identified, belonging to high frequency cells such as keratinocytes. The products of these genes take part in biological processes such as glycolysis, chromatin nuclear structure, as well as protein synthesis, degradation, and processing. Recently, Devos et al. performed laser capture microdissection to obtain separate dermis and epidermis samples [80]. This approach has partially countered the analysis difficulties due to the heterogeneity of the skin cell populations, and it makes it possible to provide information specific to the epidermis or the dermis. This work shows a disorder mainly in the epidermis and suggests a greater involvement of keratinocytes through the downregulation of *TNFAIP3*. However, the separation of the dermis and the epidermis does not prevent the challenges caused by the heterogeneity of skin cells. Indeed, skin biopsies include a significant infiltration of leukocytes which can impact the results of the transcriptome. More recently, Pascali et al. sorted CD45^neg^ epidermal cells to counter the effect of cellular heterogeneity of skin biopsies, focusing their study on keratinocytes [81]. This sorting made it possible to specifically demonstrate the keratinocytes’ role in psoriasis by attributing intrinsic changes to them, resulting from keratinocyte-specific gene deregulation, as well as changes in gene expression induced extrinsically by inflammatory mediators. Moreover, this interesting study also further investigated the contribution of transcription factors in psoriasis by identifying 54 of them whose expression was deregulated in psoriatic keratinocytes. Most are transcription factors known for their regulation of epidermal differentiation and inflammation (AP-1, NF-κB, SOX4, KLF4, GATA3, TRPS1, HEY2, and others). Some of them are also located in psoriasis susceptibility regions, representing a link with the genetic susceptibility and the molecular alterations in psoriatic skin [81]. Ultimately, single-cell RNA-seq allows the discernment of differences specific to a cell type in a heterogeneous cellular environment such as the skin. In this sense, an innovative study using single-cell RNA-seq by Cheng et al. revealed that keratinocytes change their transcriptional program as they move from the proliferative basal layer to terminal corneocytes, with 12% of transcripts differentially expressed between keratinocytes subpopulations [77]. This work very nicely illustrates the plasticity of cell transcriptional identities by highlighting two major keratinocytes subpopulations in psoriatic skin distinctively with non-inflamed skin, namely a mitotic fraction and a fraction that distinguishes by the up-regulation of ion channels and cell–cell communication transcripts. Although the proliferative fraction of keratinocytes is generally attributed to the basal layer, analyses of cell cycle related transcripts against the expression of *KRT10* suggest that they remain considerably active in the suprabasale layers of psoriatic skin, thereby confirming the proliferative nature of psoriatic keratinocytes.

### 4.3. Investigation of Treatments on the Gene Expression Profile

Further studies have been evaluating the impact of treatments for patients with psoriasis on the pattern of gene expressed by the diseased skin keratinocytes [59,73]. This approach can potentially identify genes playing a major role in the disease’s progression in comparison to those whose expression change is minimal following the administration of the treatment. Indeed, Oestreicher et al. conducted the first genome-wide study revealing the impact and influence of an immunomodulatory cytokine (recombinant human IL-11) or an immunosuppressant drug (Cyclosporin A) on gene expression. This pharmacogenomic mRNA expression analysis approach revealed a difference in the modulation of expression of various genes depending on the treatment received. Indeed, recombinant human IL-11 therapy, a treatment that reduces the production of proinflammatory cytokines such as IFNγ, IL-1β, IL-12 p40 and TNFα, resulted in the enhanced expression of genes such as *GATA3*, *CRIP1* and *TNXA* [84,85]. Cyclosporin A therapy aims to specifically inhibit calcineurin, a serine-threonine phosphatase required for the activation of the nuclear factor of activated T cells (NFAT) transcription factor, and therefore the subsequent production of IL-2 [86]. Treating psoriatic patients with cyclosporin A significantly reduced MMP-12, CCNF and HBP17 expression levels [59]. These two treatments (IL-11 and cyclosporin A) are mechanistically distinct, particularly regarding the p38/JNK signalling pathway that is impacted by Cyclosporin A, but not rhIL-11 whose mechanism appears to be due to inhibition of NF-κB nuclear translocation [87,88]. Thus, this approach makes it possible to differentiate the treatments at the gene expression level and to highlight various potential pathways for therapeutic intervention. Furthermore, Ahn et al. showed that biological treatment with adalimumab, a TNF-α inhibitor, promotes normalization of disturbed pathways of psoriasis [73]. This first study of adalimumab’s impact on gene expression network also revealed that no inversion in gene expression was observed after treatment for the control groups, suggesting an action limited to pathological skin. Therefore, differential expression analysis can identify individual genes that are differentially expressed between psoriatic cases and controls. Thus, the integration of complementary methods to biology approaches such as weighted gene co-expression network analysis with differentially expressed analysis has significant benefits over differentially expressed analysis alone. Overall, approaches studying the effects of treatments at the transcriptomic level will help define genes linked to pathogenetic processes and will broaden the understanding of psoriasis’ molecular basis.

### 4.4. Non-Lesional Skin Provides Important Insights about the Pathomechanism of Psoriasis

Additional expertise regarding the involvement of non-lesional psoriatic skin has also emerged from several transcriptomic studies [59,60,62,63,65,66,67,68,75,80,81]. Although non-lesional skin surrounding psoriatic lesions is considered to exhibit no morphological signs and is usually histologically normal, multiple studies have revealed the importance of non-lesional psoriatic skin, or psoriatic patients’ healthy skin, as well as gene expression profiles and their contribution in the development of full-blown psoriatic skin. Indeed, Gudjonsson et al. reported major changes in the non-lesional skin’s transcriptome, showing an organized gene expression system with prevailing downregulation of lipid biosynthetic genes [67]. These findings suggest that non-lesional skin might exist in a ‘‘pre-psoriatic state’’. Healthy skin can also manifest the same coordinated downregulation of lipid biosynthetic genes observed in non-lesional skin, albeit less frequently. Such analyses revealed a distinct gene expression signature for lesional skin that significantly varies from that of non-lesional or healthy skin, while the disparity between these last two is much slighter. These organized abnormalities may be linked to reduced expression of three transcription factors, including *PPARA* encoding PPAR-a, *ESR2* encoding estrogen receptor 2, and *SREBF1* encoding sterol regulatory element-binding transcription factor 1. The authors also confirmed significant dysregulation of almost all genes reported by Romanowska et al. to be abnormally expressed in lesional versus non-lesional skin and related to fatty acid signaling [67,89]. More specifically, *ELOVL3*, a part of a strongly conserved group of microsomal enzymes involved in the production of long-chain fatty acids [90], was the most significantly downregulated of the lipid biosynthetic gene transcripts. It was also downregulated in the normal skin versus psoriatic patients’ skin. Therefore, dysregulation in the expression of certain genes in non-lesional skin can often be noticed as a predisposing signature for the disease. To thoroughly investigate the role of non-lesional skin, imminent research should focus on the concept of psoriasis susceptibility genes. These new insights would determine whether any of these psoriasis susceptibility genes are involved in the biological mechanisms underlying changes in the coordinated gene expression program between non-lesional skin and healthy skin.

### 4.5. Comparison of the Most Deregulated Genes between Studies

Comparing multiple transcriptomic studies using divergent platforms and experimental conditions can be challenging. For a better overview, Table 2 displays genes emerging as being the most up- and down-regulated between healthy skin and lesional psoriatic skin from the studies in which the results were presented. Interestingly, a strong recurrence of the top upregulated genes (*S100A9, SERPINB4, PI3, S100A7, DEFB4, SERPINB3, SPRR2A, TCN1, c10orf99, AKR1B10,* and many others) is observable between studies, with an average of 75% of genes in common (Table 2). The DEGs which appear recurrently between studies participate mainly in biological functions taking part in the epidermis development, immune responses as well as in the humoral response mediated by antimicrobial peptides (Supplementary Table S1). However, the study published by Kulski et al. did not display the same similarities: a recurrence of 5% among the top upregulated genes, and no recurrence of the top downregulated were highlighted in comparison to other studies (Table 2). This low recurrence rate among the top deregulated genes emerging can be explained since the included patients were Japanese, whereas enrolled patients from other studies were mainly Caucasians. Indeed, various population-specific differences in the HLA alleles associated with psoriasis emerge from studies comparing Caucasians to Asians [91,92]. It appears that the prevalence of the causal allele HLA-C*06:02 is lower in eastern populations, and that HLA-A*02:07 is most strongly associated with psoriasis in these populations [92,93]. From this perspective, the Japanese population may exhibit unique characteristics that could also be reflected in a distinct gene expression profile. The analysis of upregulated DEGs in the study by Kulski et al. revealed some biological processes distinct from those observed in the other studies. The proteins encoded by these genes participate in protein transport besides being involved in ubiquitination and in signaling pathways, including MAPK and JAK-STAT signaling cascades (Supplementary Table S2).

### 4.6. Lipid Disturbance in Psoriasis

The skin is characterized by a rich lipid metabolism, which plays an essential role in its homeostasis [94]. Disturbed lipid metabolism is considered an important factor in the etiopathogenesis of psoriasis and it is associated with disturbed gene expressions of the enzymes required for lipid synthesis, degradation, and transport within the cells [95]. Interestingly, several genes among those that stand out as the most deregulated in psoriasis in Table 2 are associated with lipid metabolism (*AKR1B10, FABP5, ELOVL, DGAT, FADS1, GBA, GM2A, SULT2B1*). To deepen these observations, an investigation was carried out concerning the differentially expressed genes whose products are involved in lipid metabolism (Supplementary Table S3).

Major deregulation in the expression of genes that encode proteins involved in the polyunsaturated fatty acid metabolism, such as the linoleic and arachidonic acid metabolism, is characteristic of psoriatic skin [67,96]. The phospholipase A2 (PLA2) gene family members (*PLA2G4D, PLA2G4E, PLA2G2F, PLA2G4B, PLA2G2A*) are significantly overexpressed in psoriasis [96]. PLA2 are potential regulators of epidermal hyperproliferation as they hydrolyze fatty acids in position *sn*-2 of phospholipids such as linoleic and arachidonic acid, which are then metabolized into bioactive lipid mediators [97]. Consistent with higher PLA2 gene expression, levels of lipid mediators with pro-inflammatory properties including 12-HETE, LTB_4_ and PGE_2_ are enhanced in skin lesions [98,99]. These lipids are involved in keratinocyte hyperproliferation and immune cell chemotaxis [100]. Moreover, the *FADS1* and *FADS2* genes were found deregulated in psoriatic skin [67]. The delta-6-desaturase and delta-5-desaturase, encoded by the *FADS2* and *FADS1* genes, respectively, perform the first and the last steps of the pathway in which linoleic acid is converted into arachidonic acid and are key enzymes regulating the metabolization of polyunsaturated fatty acids [101,102]. The gene coding for the aldo-keto reductase family 1 member 10 (*AKR1B10*,), was the highest DEG expressed in psoriatic tissue compared to normal tissue according to Gao et al. [96]. This gene is part of the linoleic acid metabolism, which also implies a malfunction of this lipid pathway in psoriasis pathogenesis. The knockdown of *ARK1B10* showed its involvement in cellular growth as well as lipid synthesis, particularly phospholipids [96].

Important proteins involved in the establishment of the skin barrier were also found among the most deregulated genes in psoriasis. Long chain fatty acid elongases 3 (ELOVL3), encoded by *ELOV3L*, were found to be downregulated in psoriasis [67]. ELOVL3 catalyzes the elongation of fatty acids composed of a chain of 20 carbons (C20) to C22 or C24 fatty acids and plays an important role in the lipid matrix organization of the stratum corneum [103]. In addition, the fatty acid binding proteins (FABPs) are proteins encoded by *FABP* genes whose contribution to psoriasis has been pointed out only lately (*FABP5* and *FABP7*) [64,67]. *FABP5* knockout in mice display decreased barrier function recovery after disruption as well as impaired keratinocyte motility [104]. Finally, *DGAT2L6* expression was also found deregulated in psoriasis [72]. This gene codes for diacylglycerol acyltransferase 2 (*DGAT2*) that catalyzes the final step of triglyceride synthesis [103]. The *DGAT2* knockout mice have defects in permeability barrier functions that are lethal soon after birth [105].

### 4.7. Associations between Transcriptomic Profiles in Different Conditions

The use of comparative transcriptomic studies is interesting in understanding the mechanisms governing different contexts or diseases. First, Mee et al. demonstrated the potential participation of IL-1 in psoriasis by comparing the transcriptome of IL-1-induced keratinocytes to that of the psoriatic skin, suggesting a dominant immune response in the inflammatory environment of psoriatic lesions rather than an adaptative immune response [64]. Comparative transcriptomic analyses can also be useful in establishing the differences between psoriasis subtypes. Indeed, Ahn et al. investigated the effect of anatomic location in psoriasis subtypes, such as the scalp, palmoplantar, and conventional plaque psoriasis. Their study revealed the molecular heterogeneity of plaque psoriasis in addition to identifying subtype-specific signaling pathways [76]. This valuable information can then help develop appropriate therapies for plaque psoriasis subtypes.

Another utility of using transcriptomic studies is to compare the differences between pathologies with similarities to better define their specific molecular bases. In this sense, several research teams have been interested in investigating the differences between atopic dermatitis (AD) and psoriasis, two immune-mediated skin diseases. Nomura et al. were the first to identify a distinctive pattern of gene expression that characterizes AD versus psoriasis [61]. The most marked differences involve the expression of various chemokines. Indeed, expression of genes encoding CC chemokines such as *CCL13*, *CCL18* and *CCL27* was found as being increased in AD skin while that of *CCL4*, *CCL20*, *CXCL2*, *CXCL8* and *CXCR2* was rather increased in psoriatic skin. From this identified chemokine deregulation, further links were established allowing the clarification of the distinct leukocyte infiltration pathways of these two skin diseases.

A few years later, a study by Nattkemper et al. focused on the investigation of gene expression linked to itching in the skin of patients with AD and psoriasis [78]. This was the first study examining gene expression specifically linked to chronic itch. A gene signature associated with itching was highlighted by comparing the gene expression profiles of skin from AD or psoriatic patients with itch severity scores. Although differences could be observed between itch-associated mediators and receptors in AD and psoriasis, a common set of deregulated genes has been identified, including *PLA2G4B, PLA2G4D, PLA2G4E*, *TRPV1*, *TAC1, TACR1, TPSAB1, HRH2, KLK6, KLK14, S100A9*, as well as genes encoded for various chemokines. These valuable data could be useful in establishing effective treatments for the relief of itching in pruritic skin.

More recently, Devos et al. reported a decrease in *TNAIP3* gene expression, a negative regulator of NF-κB, that is also named A20, in the human epidermis [80]. According to their results, the absence of A20 in keratinocytes led to systemic inflammation and is proved sufficient to exacerbate both AD and psoriatic immune profiles by increasing the expression of cytokines and chemokines. While there are some commonalities between these two skin conditions, the dichotomy of chemokines profiles found in AD versus psoriasis could be a key factor in the control of chronic skin inflammation.

### 4.8. Factors to Consider When Comparing Transcriptomic Studies

Several factors should be taken into consideration when analyzing transcriptomic studies. Firstly, interindividual variability is an important cause of the discrepancy between studies. Indeed, intrinsic genetic factors, as well as other extrinsic elements, including age, pharmacological treatments, and other environmental conditions can have a significant impact on gene expression [106]. A better understanding of molecular heterogeneity is still needed to better understand the molecular function and molecular noise. To reduce the differences caused by interindividual variability, a large number of biological samples is needed, especially in the case of complex studies such as transcriptomic analysis. Secondly, the microarray technique uses levels of hybridization quantified using fluorescence that is converted into expression measurements. An undeniable consequence is a low reproducibility between laboratories because of the change in fluorescence, representing the hybridization intensities, measured between different laser scanners. The MicroArray Quality Control consortium was developed to address this concern, as well as other performance and data analysis issues [107]. It is now considered to be the gold standard method for performing reproductible and accurate measurements as well as for confirming the results from high-throughput platforms [108]. Finally, another possible source of discrepancy is the batch effect, a common source of technical variation that may then be interpreted as a biologically significant finding [109]. Pairs of non-lesional and lesional skin samples might not always be processed simultaneously, thus creating a batch effect inflating the number of differently expressed genes. This confusing effect can be avoided by concurrently running the paired non-lesional and lesional skin samples [68].

It also remains important to recognize the differences between the available technologies for transcriptome analysis. The main difference between microarrays and RNA-seq is that the latter allows complete sequencing of the transcriptome. Indeed, while microarrays are effective at identifying known genes and transcripts, the information they provide is incomplete as this transcriptomic analysis procedure does not allow the identification of previously unidentified genes [110]. In contrast, RNA sequencing enables gene expression to be reliably detected and quantified even when genes are expressed at low levels [72]. This technique is now a popular tool for studying gene expression and the discovery of novel RNA species. Nevertheless, microarrays offer some advantages over RNA-seq. The main disadvantages of RNA-seq are the lack of an accurate algorithm for analysis as well as the size of the data folder, thus providing a challenge for their analysis [111,112]. Indeed, due to potential sequencing, alignment and annotation errors, data processing and analysis become challenging, and the development of more accurate algorithms has been suggested [111]. Finally, the high variability of data collected by single-cell RNA-seq also raises computational challenges in data analysis [113]. An additional challenge with single-cell RNA-seq lies in the analysis of cells that are found to be excluded during data processing. This applies particularly to skin cells since the terminal keratinocytes, i.e., the corneocytes, are dying cells. Thereby, this cell type is then excluded during flow cytometry 4’-6-diamidino-2-phenylindole (DAPI)-negative gating, the aim of which is to eliminate the DAPI-positive dead cells from the analysis [77].

## 5. Transcriptomic Studies Using In Vitro Models

Psoriatic skin biopsies are very useful when studying alterations in gene expression since their analysis allows the identification of genes whose expression is limited to specific skin cell types [114]. However, this model is still not widely available or accessible. Consequently, a few research teams developed psoriatic-like keratinocytes in monolayer as a tool for studying the participation of keratinocytes in psoriasis [115,116,117]. Muromoto et al. have attempted to mimic the inflammatory environment found in psoriatic lesions by treating the epidermal keratinocytes of healthy human skin in monolayer with a set of cytokines specific to the pathology, including IL-17A, TNF-α, IL-17C, IL-22, IL-36γ and IFN-γ, to study the changes induced on the transcriptomic profile [115]. Interestingly, such addition of psoriatic-specific cytokines during the culture of keratinocytes made it possible to study the different genes deregulated at the level of keratinocytes for each of the cytokines involved [57]. Although it was possible to compare the transcriptomic profile of psoriatic-like keratinocytes to that from psoriatic skin biopsies, the fact remains that several elements are missing from this type of study to make an adequate comparison to in vivo skin, namely fibroblasts, immune cells, nerve components, and the presence of an extracellular matrix [64].

Gudjonsson et al. recently compared the transcriptome of both lesional psoriatic skin and cultured keratinocytes previously stimulated by a set of key cytokines similar to previous studies, namely IL-17, IL-22, IL-1a, IFN-g, TNF-α, in addition to oncostatin-M to determine whether or not the deregulated gene expression profile of the cultured cytokine-stimulated keratinocytes also reflects that of the lesional psoriatic skin. In this sense, the genes from the transcriptomic profile of cultured cytokine-stimulated keratinocytes overlapping with that of the lesional psoriasis skin only represented up to 5.0% of the psoriatic altered transcriptome [68]. Wang et al. observed similar results regarding the proportion of deregulated genes overlapping between cultured cytokine-stimulated keratinocytes and lesional psoriatic skin while using HaCat keratinocytes, and thus showed once again the limitation of using monolayer systems to obtain a gene alteration profile that mimics the pathology in the most representative manner [118]. The lack of pathological keratinocytes in studies using these monolayer psoriatic-like models represents a major disadvantage for further transcriptomic studies.

In contrast to the limitations of healthy keratinocytes’ culture, the monolayer culture of psoriatic keratinocytes has proven to be a useful method when examining the intrinsic defects of pathological keratinocytes without the involvement of external factors. Indeed, a study conducted by Swindell et al. compared the deregulated genes of a monolayer psoriatic keratinocyte model with those of full-thickness psoriatic skin lesions to determine whether changes in the transcriptomic profile are due to intrinsic changes in keratinocytes or are representative of the keratinocyte response to their environment [75]. Their results showed that differences in the profile of deregulated genes were greater when comparing both healthy and psoriatic keratinocytes grown in monolayer model together than when comparing healthy and psoriatic full-thickness skin. This highlights the fact that analysis of keratinocytes grown in monolayer could possibly detect deregulated genes that could be specific to keratinocytes in contrast to analysis of full-thickness biopsies. Indeed, their team identified 300 genes that were deregulated for keratinocytes grown in monolayer in contrast with a difference of only 39 genes deregulated for full-thickness skin. Thereby, their team was the first to identify a considerable number of downregulated genes significantly overlapping with the transcriptomic profile of previous psoriasis studies, especially at the level of genes associated with epidermal differentiation. Therefore, although these observations may in part come from the cell culture condition itself, such important changes may also provide new gene alterations specific to pathological keratinocytes that could not have been revealed in the past due to the multicellular context of the models used [75].

Considering the limitations of the monolayer systems, the employment of more complex and stratified models is expected to provide further information on the effect and the role of key cytokines on the pathogenesis and thereby, on the pathology of psoriasis itself [119]. Indeed, although monolayer psoriatic models are very useful to study deregulated genes associated with pathological keratinocytes, this type of model does not provide a transcriptomic profile that highlights all the different interactions existing between the different components of psoriatic skin, hence the interest in 3D models for transcriptomic studies. Interestingly, several 3D models have been developed using different matrices and cell types to mimic the microenvironment found in psoriatic skin [120]. First, Barker et al. developed a 3D psoriatic model derived from both psoriatic keratinocytes and fibroblasts to compare the phenotypic characteristics to the same model made of healthy cells [121]. This collagen-based psoriatic skin model, assembled both from lesional and non-lesional cells, exhibits a higher proliferation rate than the healthy model, as well as increased production of TNF-α, IL-8 and IFN-γ. Our team has developed a similar approach without using collagen gel as a matrix [122]. Indeed, our model relies on the ability of fibroblasts to secrete their own extracellular matrix under well-defined conditions, thereby eliminating the need for exogenous material. We also showed that the combination of both psoriatic keratinocytes and psoriatic fibroblasts has a significant impact on the development of a psoriatic model closest to the physio-pathological characteristic of psoriasis [123].

This psoriatic model was then used for gene profiling analysis to compare the gene expression profile of substitutes made from healthy and non-lesional psoriatic cells with those made up of cells isolated from the pathological lesions [124]. The results of that study showed significant differences in gene expression between healthy and lesional substitutes. These differences are significantly less important when the altered genes of non-lesional cells are compared with those of lesional cells, even though the cells are from the same patient, suggesting a change in the expression of some genes upon transition from non-lesional cells into lesional cells. The identification of different gene profiles between healthy cells from healthy donors and healthy cells from patients with psoriasis is a very interesting result that emerged from this transcriptomic study, supporting the interest on the roles of non-lesional cells in the pathogenesis of psoriasis. Furthermore, the comparison of the data files from this study with those of Gudjonsson et al. and Oestreicher et al. revealed that 54% of the deregulated genes are common between both studies [124]. Considering that this psoriatic skin model contains only psoriatic fibroblasts and keratinocytes whereas the psoriatic skin contains all the different skin cell types is encouraging in that it demonstrates the reliability of this in vitro model.

From a tridimensional model including pathological cells, it is hence possible to add different psoriasis related components such as cytokines, dendritic cells, etc. to complexify the model itself, and thus obtain additional responses to those yielded solely by using both psoriatic keratinocytes and fibroblasts. In this sense, our team also supplemented the tissue-engineered human psoriatic skin model with different cytokines, namely TNF-α, IL-1α, IL-6 and IL-17A intending to identify variations in the transcriptome that occurred consequently to the addition of these cytokines. The addition of this cytokine cocktail led to a pattern of expressed genes much closer to that of the native psoriatic skin. Indeed, various genes such as *S100A12*, *CXCL8*, *DEFB4A*, *KYNU, CCL27, ACSBG1* and *SERPINA12* are genes that have already been reported to be deregulated in the psoriatic skin, and their expression was not found deregulated in the control psoriatic substitutes, i.e., without addition of cytokines [125]. These results demonstrate the interest in developing complex 3D models comprising a variety of pathological cell types in order to obtain gene expression profiles closer to those that are typical of in vivo skin.

To conclude, 3D skin models offer a tremendous potential for the study of skin disease. These models can also serve as a platform for personalized medicine. Indeed, with a simple skin biopsy, several skin substitutes can be reproduced in vitro. These substitutes are then available for a variety of analyses and therefore make it possible to quickly find effective therapeutic targets for a specific patient.

## 6. Conclusions

Although the basic knowledge of psoriasis increased throughout recent years, the understanding of its molecular basis has yet to be clarified. Differential gene expression profiles allowed the development of new hypotheses to further explain the underlying pathology of this multi-genic skin disease. Some evidence suggests that the initiation and progression of psoriasis likely originate from the keratinocytes, where an outburst of endogenous inflammatory responses contributes to adaptive immunity. It remains unclear whether keratinocytes’ or the immune system’s abnormalities cause the pathogenesis of psoriasis. However, keratinocytes undoubtedly play an important role in the development of psoriasis as well as in the initiation of cutaneous inflammation. Recognition of the importance of the keratinocytes’ role raises new questions and directions for future treatments of this disease and the development of effective medications.

## Figures and Tables

**Table 1:** Psoriasis transcriptomic analyses.

Year	References	Samples	Key Message	Methodology and Validation
2001	Oestreicher et al. [59]	24 psoriatic skin biopsies (lesional and non-lesional)	This first genome-wide analysis following a pharmacological intervention has shown that treatment can influence the effect on gene expression. Recombinant human IL-11 treatment resulted in increased expression of genes such as *GATA3*, *CRIP1* and *TNXA*, while cyclosporin A significantly decreased the levels of *MMP12*, *CCNF* and *HBP17*.	Microarray (HuGeneFL, Affymetrix Inc, Santa Clara, CA, USA)
2001	Bowcock et al. [60]	15 psoriatic skin biopsies (lesional and non-lesional)	Hierarchical clustering analyses showed a strong difference in gene expression between lesional and non-lesional samples, particularly genes expressed by inflammatory cells. These variances are independent of the HLA-Cw*0602 status of the patients.	Microarray (HG-U95A, Affymetrix Inc, Santa Clara, CA, USA)
2003	Nomura et al. [61]	Skin biopsies from 7 patients with psoriasis and 6 patients with atopic dermatitis (AD)	Study showing for the first time the distinctive pattern of gene expression that characterizes AD versus psoriatic skin lesion. The 18 genes whose expression is increased in AD skin, including the CC chemokines *CCL13*, *CCL18*, and *CCL27*, as well as the 62 genes whose expression is significantly increased in psoriatic skin compared to AD skin, including *CCL4*, *CCL20*, *CXCL2*, *CXCL8*, and *CXCR2*, providing a potential signature for the identification of these skin diseases.	Microarray (HG-U95Av2, Affymetrix Inc, Santa Clara, CA, USA) and **qRT-PCR**
2003	Zhou et al. [62]	Psoriatic skin biopsies (lesional and non-lesional)	Identification of two new genes in the IL-1 family genes products that are deregulated in psoriatic lesions, namely *IL1HY1* and *IL1H1*. Ontology analyses have also revealed that epidermal differentiation is the only constantly altered process between lesional/non-lesional skin and normal skin.	Microarray (HG-U95A-E, Affymetrix Inc, Santa Clara, CA, USA) and **qRT-PCR**
2005	Kulski et al. [63]	4 psoriatic skin biopsies (lesional and non-lesional)	Gene expression profiling of Japanese psoriatic skin identified up-regulated genes involved in atypical epidermal cellular organization and differentiation, such as *TGM1*, *IVL*, *FABP5*, *CSTA* and *SPRR*. *JUNB* stands out as the most deregulated gene between lesional and non-lesional samples.	Microarray (HG-U95Av2, Affymetrix Inc, Santa Clara, CA, USA)
2007	Mee et al. [64]	6 psoriatic skin biopsies and healthy keratinocytes culture treated with cytokines	A significant correlation between upregulated transcripts in psoriatic skin lesions and the ones from IL-1-induced keratinocytes in vitro suggests a dominant innate immune response in the inflammatory environment of psoriatic lesions rather than an adaptive immune response.	Microarray (HuGeneFL and HG-U133A, Affymetrix Inc, Santa Clara, CA, USA)
2007	Reischl et al. [65]	20 psoriatic skin biopsies (lesional and non-lesional)	Increased expression of *WTN5A* in psoriatic lesions. The involvement of Wtn5a in the pathogenesis of psoriasis remains to be elucidated but increased signaling via Wtn5a could lead to the activation of phospholipase C, and subsequently to the stimulation of the protein kinase C and calcineurin pathways.	Microarray (HG-U133A, Affymetrix Inc, Santa Clara, CA, USA) and **qRT-PCR**
2008	Yao et al. [66]	26 psoriatic skin biopsies (lesional and non-lesional)	Type I IFNs as well as *IFNAR1* and *IFNAR2* are overexpressed in psoriatic lesional skin. The presence of a signature corresponding to induction by type I IFNs in lesional psoriatic skin suggests active signaling in these lesions.	Microarray (HG-U133 Plus 2.0, Affymetrix Inc, Santa Clara, CA, USA) and **qRT-PCR**
2009	Gudjonsson et al. [67]	58 psoriatic skin biopsies (lesional and non-lesional)	Non-lesional skin could exist in a “pre-psoriatic” state. The decrease in the activity of three transcription factors, namely *PPARA*, *ESR2* and *SREBF1*, could be linked to the altered expression of genes involved in lipid metabolism.	Microarray (HG-U133 Plus 2.0, Affymetrix Inc, Santa Clara, CA, USA) and **qRT-PCR**
2010	Gudjonsson et al. [68]	58 psoriatic skin biopsies (lesional and non-lesional)	A large-scale study identifying more than 600 previously unreported transcripts. The comparison of this study with the cytokine-stimulated keratinocytes transcriptomes showed moderate overlap with the psoriatic transcriptome, without however representing more than 5.6% of the complete psoriatic transcriptome.	Microarray (HG-U133 Plus 2.0, Affymetrix Inc, Santa Clara, CA, USA) and **qRT-PCR**
2011	Joyce et al. [69]	Psoriatic skin biopsies (lesional and non-lesional)	Identification of a variety of new microRNAs by RNA-sequencing (RNA-seq). Of interest are an uncharacterized keratinocyte-derived miRNA, miR-135b, as well as an epidermal infiltration of the specific hematopoietic miRNA, miR-142-3p, in psoriatic lesions. The functions of these newly identified miRNAs are consistent with the inflammatory and hyperproliferative features of psoriasis.	Illumina (GAIIx, Illumina, San Diego, CA, USA) and **qRT-PCR**
2012	Suarez-Farinas et al. [70]	85 psoriatic skin biopsies (lesional and non-lesional)	A study providing a complex molecular definition of moderate-to-severe psoriasis. Ingenuity pathway analysis identified activated transcription factors, such as *NROB2*, whose function is the downregulation of target genes. This could also explain the PPARa and RAR activation.	Microarray (HG-U133 Plus 2.0, Affymetrix Inc, Santa Clara, CA, USA) and **qRT-PCR**
2012	Jabbari et al. [71]	Psoriatic skin biopsies (lesional and non-lesional)	The comparison of DEGs obtained from this study carried out by RNA-seq with those which used microarray technologies revealed a substantial number of previously unrecognized DEGs in psoriatic skin. The data obtained supported the importance of the synergy of the combined action of IL-17 and TNF-α in the pathogenesis of psoriasis.	RNA-seq (GAIIx, Illumina, San Diego, CA, USA)
2014	Li et al. [72]	92 psoriatic skin biopsies	RNA-seq has put forward the involvement of transcripts with low expression levels which could not be identified by microarray studies by revealing gene coexpression networks illuminating processes involving keratinocytes, myeloid cells and T cells.	RNA-seq (GAII, Illumina, San Diego, CA, USA) and microarray (HG-U133 Plus 2.0, Affymetrix Inc, Santa Clara, CA, USA)
2016	Ahn et al. [73]	18 psoriatic skin biopsies (pre- and post-treatment with adalimumab)	Treatment with a TNF-α inhibitor, such as adalimumab, promotes normalization of disturbed pathways of psoriasis. The top enriched pathways after biological treatment are the regulation of leukocyte mediated cytotoxicity, regulation of cell killing and leukocyte activation. This study also highlights several roles for long non-coding RNAs.	RNA-seq (HiSeq 2000, Illumina, San Diego, CA, USA)
2016	Swindell et al. [74]	Psoriatic skin biopsies (performed using reads from prior studies)	The psoriasis specificity index developed revealed that psoriasis specific DEGs are mainly expressed by keratinocytes. Moreover, the activation of the IL-17A pathway appears to be a unique characteristic of psoriasis lesions and an inducer of DEGs.	RNA-seq meta-analysis (GSE41745, GSE54456/GSE63979 and GSE66511)
2017	Swindell et al. [75]	Psoriatic keratinocyte monolayer culture compared to 4 skin biopsies from the same patients (lesional and non-lesional)	Identification of a decrease in the expression of early and late differentiation markers (*KRT1*, *KRT10*, *FLG*, *LOR*) as well as of differentiation mediators (*CAP14*, *ACER1*) in psoriatic monolayer keratinocytes compared to full-thickness psoriatic biopsies, proposing differentiation as a key dysregulated process. A few genes under-expressed in the psoriatic keratinocytes monolayer have been associated with AP-1 binding sites located in the region of the epidermal differentiation complex, suggesting an intrinsic alteration in psoriatic keratinocytes.	RNA-seq (TruSeq mRNA Sample Prep v2 kit, Illumina, San Diego, CA, USA)
2018	Ahn et al. [76]	Biopsies from scalp (8), palmoplantar (2) and conventional (8) plaque psoriasis	RNA-seq analysis identifies a core set of DEGs common to the scalp, palmoplantar and conventional psoriasis, including genes involved in the activation and proliferation of keratinocytes, such as *S100A7A*, *SPRR2A/B*, *SERPINB4*, *S100A9*, *KRT6*, *C10orf99*, *LCE3D/E* and *IL36G*. The psoriasis subtypes show a differential expression level of the DEGs in this core set and displayed differences in IL-17A, IFNγ, and IL-22 production.	RNA-seq (HiSeq, Illumina, San Diego, CA, USA) and flow cytometry
2018	Cheng et al. [77]	9 normal and 3 psoriatic epidermis manually separated from the dermis	Characterization of epidermal cell state according to three anatomical sites (scalp, trunk, and foreskin) as well as the psoriatic epidermis using single-cell RNA-seq revealed a discrete set of specialized keratinocytes that exhibit a distinct composition at different anatomic sites.	Single-cell RNA sequencing (HiSeq 2500, HiSeq 4000 or NovaSeq 6000, Illumina, San Diego, CA, USA)
2018	Nattkemper et al. [78]	25 psoriatic skin biopsies	Identification of a gene signature associated with itching is described as “itchscriptome”. These genes are expressed by skin cells, immune cells, and nerves. Their products encode mediators and receptors associated with all aspects of the transmission of itching at the peripheral level.	RNA-seq (NextSeq, Illumina, San Diego, CA, USA)
2018	Qiao et al. [79]	13 psoriatic skin biopsies	Results shed light on the effect of deregulation of circRNA expression and its potential biological functions in regulating immunity, inflammation, and proliferation of psoriasis. The aberrant expression of has_circ_0061012, a circRNA with five miRNA binding sites, may be involved in psoriasis and may be a potential biomarker.	Microarray (human ceRNA array V1.0 4x180K, Shanghai Biotechnology Corporation, Shangai, China) and **qRT-PCR**
2019	Devos et al. [80]	9 psoriatic skin biopsies (lesional and non-lesional, dermis and epidermis)	*TNAIP3* is decreased in the human epidermis, but not in the dermis, suggesting that down-regulation of *A20/TNFAIP3* is likely due to a decrease in its expression by keratinocytes. IMQ-induced psoriatic A20-deficient mice show that skin inflammation caused by loss of A20 in keratinocytes drives systemic inflammation, thus offering a promising therapeutic target.	Microarray (MDS Analytical Technologies, Sunnyvale, CA, USA)
2019	Pascali et al. [81]	Keratinocytes enriched from 9 psoriatic skin biopsies (lesional and non-lesional)	Gene set enrichment analysis highlighted the dominance of an IL-22/IL-17A signature in psoriatic keratinocytes transcriptome. MetaCore analysis of DEGs revealed significantly enriched sites for multiple transcription factors. Among these, transcription factors still poorly characterized in the context of psoriasis have been identified, namely *TRPS1* and *HEY2*. This study emphasizes the major contribution of keratinocytes in the molecular changes in psoriasis.	Microarray (HTA 2.0, Affymetrix Inc, Santa Clara, CA, USA) and **qRT-PCR**

DEG, differentially expressed genes, the bold highlights studies that confirmed their gene profiling data.

**Table 2:** Most up- and down-regulated genes between healthy and lesional psoriatic skin.

	Methodology	Top Upregulated Genes	Top Downregulated Genes
Bowcock et al. [60]	Microarray (Affymetrix)	*S100A2, S100A7, S100A8, S100A9, SPRR2A, SPRR1B, SPRK, CSTA, FABP5, DEFB2, KRT6A, IFI27, KRT16A, KRT17, SERPINB3, SERPINB4, PI3*	*KRT15, JUND, XP5, TNA, CRIP1, COL1A2, GSN, PCBP2, LGALS3, HBA1, MT1L, DF, LGALS1*
Kulski et al. [63]	Microarray (Affymetrix)	*JUNB, YWHAB, LAMP3, SEC61G, KIAA0101, CSTA, OAS1, CCL20, TGM1, SEC61B, GBA, H2AFY, UBE2L6, GM2A, SULT2B1, P4HB, RER1, PSMB6, NMI, IVL*	*CSPG4, ANP32A, SPTAN1, C11orf11, SSA2, LAMA5, ICA1, MECP2, ALDH3A2, NAB1, DDHD2, ANXA8, ZNF384, OSBPL1A, ZBTB16, ATXN3, DDR1, GPM6B, KCNC1, PCDH21*
Mee et al. [64]	Microarray (Affymetrix)	*SERPINB4* *, PI3, S100A9, S100A7, DEFB4, KRT6A, SERPINB3, SPRR2B, KRT17, KRT16, SPRR2A, SPRR2D, GJB2, KRT6E, KRT6B, CSTA, SPRR1B, TCN1, SPRR1A, CD24, LCN2, SPRR2E, FABP5*	*HBB, HBA2, KRT2A, ZNF91, MUC5B, MT1X, GATA3, LOR, ACTA2, SFTPA2, CST6, TXNIP, MBP, LGALS3, MUC6*
Gudjonsson et al. [67]	Microarray (Affymetrix)	*C10orf99* *, SPRR2B, S100A7, LCE3D, SPRR2G, WFDC12, S100A9, HAL, IL1F9, DEFB4*	*ELOVL3* *, FLJ32569, HSD3B1, MLSTD1, GAL, KRT6L, THRSP, FADS1, MUC7, SCGB2A1*
Gudjonsson et al. [68]	Microarray (Affymetrix)	*SERPINB4* *, DEFB4, S100A7L1, PI3, SERPINB3, SPRR2C, AKR1B10, S100A12, S100A9, C10orf99, KYNU, LCE3D, S100A7, CXCL8, KRT16*	*WIF1* *, BTC, THRSP, IL1F7, CCL27, KRT1B, MSMB, ELOVL3, GAL, FABP7, ACSBG1, MLSTD1, HS3ST6, WDR72, SERPINA12*
Li et al. [72]	RNA-seq (Illumina)	*DEFB4A* *, S100A7A, PI3, LCE3A, S100A12, S100A9, SERPINB4, TCN1, S100A8, CXCL8, TMPRSS11D, S100A7, SPRR2F, TNIP3, SERPINB3, HEPHL1, GDA, AKR1B10, CXCL13, SPRR2A*	*AADACL3, PM20D1, DGAT2L6, AWAT2, AWAT1, PNPLA5, ROS1, GAL, CYP2W1, UGT3A2, C10orf129, PDE6A, BTC, TRIM55, WIF1, ELOVL3, HGD, HAO2, SYT9, LPPR5*
Ahn et al. [76]	RNA-seq (Illumina)	*IL36A, SPRR2F, SPRR2A, SERPINB4, S100A7A, SPRR2B, PI3, TCN1, S100A9, KRT6C, TMPRSS11D, HEPHL1, SPRR2D, IL17F, C10orf99, LCE3E, KRTAP9-7, LCE3D, AKR1B10, KRTAP13-1*	*SERTM1, IL6, ADAMTS16, CYP2W1, FOS, CSF3, BTC, C16orf89, FAM95C, MATN4, PDK4, KRT77, CILP2, UGT3A2, BMP3, NR4A1, WNT2, MAB21L1, HAS1, SERPINE1*
Pasquali et al. [81]	Microarray (Affymetrix)	*PI3* *, DEFB4A, SERPINB4, TCN1, DEFB4B, S100A9, AKR1B10, S100A7A, KYNU, SERPINB3, IFI6, SPRR2A, IFI27, OAS2, IFI44L, C10orf99, IFI44, HEPHL1, EPGN*	*FAM26E, NR4A3, KRT77, HIST2H4B, APOD, CA6, KRT31, ATF, ZNF667-AS1, TNFAIP3, C5orf46, FTL, CLDN8, CRABP1, AGR2, CDA, PPAP2A, TRAM2, CRYAB, AF212831.2*

Genes identified in red appeared in two or more studies.

## Supplementary Materials

The following are available online at https://www.mdpi.com/2073-4425/11/10/1155/s1, Table S1: Biological functions of the proteins encoded by the upregulated DEGs recurrent between transcriptomic studies, Table S2: Biological functions of the proteins encoded by the upregulated DEGs in the study by Kulski et al., Table S3: DEGs from pathways related to lipid metabolism.
