# Supplementary material for: Transcriptome Profiling Analyses in Psoriasis: A Dynamic Contribution of Keratinocytes to the Pathogenesis
Source: Genes (Basel). 2020 Sep 30;11(10):1155. doi: 10.3390/genes11101155 (PMC7600703; doi:10.3390/genes11101155)
Supplement: Supplementary file 1 [file genes-11-01155-s001.pdf]

SUPPLEMENTARY MATERIALS

Supplementary Table 1. Biological functions of the proteins encoded by the upregulated DEGs recurrent between transcriptomic studies.

Gene Name	Protein Name	Functions
S100A9	S100 Calcium Binding Protein A9, Calgranulin B	GO:0005515 protein binding; GO:0005576 extracellular region; GO:0005615 extracellular space; GO:0062023 collagen-containing extracellular matrix; GO:0005509 calcium ion binding; GO:0005737 cytoplasm; GO:0005829 cytosol; GO:0005856 cytoskeleton; GO:0005886 plasma membrane; GO:0070062 extracellular exosome; GO:0002523 leukocyte migration involved in inflammatory response; GO:0005634 nucleus; GO:0006914 autophagy; GO:0006954 inflammatory response; GO:0008017 microtubule binding; GO:0008270 zinc ion binding; GO:0030593 neutrophil chemotaxis; GO:0035662 Toll-like receptor 4 binding; GO:0045087 innate immune response; GO:0050544 arachidonic acid binding; GO:0050786 RAGE receptor binding; GO:0061844 antimicrobial humoral immune response mediated by antimicrobial peptide; GO:0070488 neutrophil aggregation; GO:0001816 cytokine production; GO:0002224 toll-like receptor signaling pathway; GO:0002376 immune system process; GO:0002544 chronic inflammatory response; GO:0005654 nucleoplasm; GO:0006915 apoptotic process; GO:0006919 activation of cysteine-type endopeptidase activity involved in apoptotic process GO:0006935 chemotaxis; GO:0007267 cell-cell signaling; GO:0010976 positive regulation of neuron projection development; GO:0014002 astrocyte development; GO:0016020 membrane; GO:0016209 antioxidant activity; GO:0018119 peptidyl-cysteine S-nitrosylation; GO:0019730 antimicrobial humoral response; GO:0030054 cell junction; GO:0030307 positive regulation of cell growth; GO:0032119 sequestering of zinc ion; GO:0032496 response to lipopolysaccharide; GO:0032602 chemokine production; GO:0034774 secretory granule lumen; GO:0035425 autocrine signaling; GO:0035606 peptidyl-cysteine S-trans-nitrosylation; GO:0035821 modulation of process of other organism; GO:0042742 defense response to bacterium; GO:0043312 neutrophil degranulation; GO:0045113 regulation of integrin biosynthetic process; GO:0046872 metal ion binding; GO:0048306 calcium-dependent protein binding; GO:0050727 regulation of inflammatory response; GO:0050729 positive regulation of inflammatory response; GO:0050832 defense response to fungus; GO:0051092 positive regulation of NF-kappaB transcription factor activity; GO:0051493 regulation of cytoskeleton organization; GO:0098869 cellular oxidant detoxification; GO:2001244 positive regulation of intrinsic apoptotic signaling pathway
SERPINB4	Serpin Family B Member 4	GO:0004867 serine-type endopeptidase inhibitor activity; GO:0005615 extracellular space; GO:0002020 protease binding; GO:0010466 negative regulation of peptidase activity; GO:0010951 negative regulation of endopeptidase activity; GO:0016020 membrane; GO:0016021 integral component of membrane; GO:0019899 enzyme binding; GO:0042270 protection from natural killer cell mediated cytotoxicity

PI3	Peptidase inhibitor 3, skin derived	GO:0004867 serine-type endopeptidase inhibitor activity; GO:0005615 extracellular space; GO:0002020 protease binding; GO:0010466 negative regulation of peptidase activity; GO:0010951 negative regulation of endopeptidase activity; GO:0016020 membrane; GO:0016021 integral component of membrane; GO:0019899 enzyme binding; GO:0042270 protection from natural killer cell mediated cytotoxicity
S100A7	S100 calcium-binding protein A7, psoriasin	GO:0005576 extracellular region; GO:0005515 protein binding; GO:0005737 cytoplasm; GO:0005509 calcium ion binding; GO:0005615 extracellular space; GO:0005829 cytosol; GO:0008270 zinc ion binding; GO:0000302 response to reactive oxygen species; GO:0001525 angiogenesis; GO:0005634 nucleus; GO:0005783 endoplasmic reticulum; GO:0005925 focal adhesion; GO:0008544 epidermis development; GO:0010820 positive regulation of T cell chemotaxis; GO:0019730 antimicrobial humoral response; GO:0030216 keratinocyte differentiation; GO:0032496 response to lipopolysaccharide; GO:0035578 azurophil granule lumen; GO:0043312 neutrophil degranulation; GO:0045087 innate immune response; GO:0046872 metal ion binding; GO:0046914 transition metal ion binding; GO:0048306 calcium-dependent protein binding; GO:0050786 RAGE receptor binding; GO:0050829 defense response to Gram-negative bacterium; GO:0051238 sequestering of metal ion; GO:0061844 antimicrobial humoral immune response mediated by antimicrobial peptide; GO:0062023 collagen-containing extracellular matrix; GO:0070374 positive regulation of ERK1 and ERK2 cascade; GO:0071624 positive regulation of granulocyte chemotaxis; GO:0090026 positive regulation of monocyte chemotaxis
DEFB4	Beta-defensin 4A	GO:0031640 killing of cells of other organism; GO:0005576 extracellular region; GO:0005615 extracellular space; GO:0006935 chemotaxis; GO:0042742 defense response to bacterium; GO:0005515 protein binding; GO:0005796 Golgi lumen; GO:0006952 defense response; GO:0031731 CCR6 chemokine receptor binding; GO:0050829 defense response to Gram-negative bacterium; GO:0050830 defense response to Gram-positive bacterium; GO:0061844 antimicrobial humoral immune response mediated by antimicrobial peptide; GO:0006955 immune response; GO:0007186 G protein-coupled receptor signaling pathway; GO:0019730 antimicrobial humoral response; GO:0042056 chemoattractant activity; GO:0050918 positive chemotaxis; GO:0060326 cell chemotaxis
SERPINB3	Serpin Family B Member 3	GO:0004867 serine-type endopeptidase inhibitor activity; GO:0005634 nucleus; GO:0005737 cytoplasm; GO:0002020 protease binding; GO:0005615 extracellular space; GO:0010466 negative regulation of peptidase activity; GO:0010951 negative regulation of endopeptidase activity; GO:0070062 extracellular exosome; GO:0001618 virus receptor activity; GO:0004869 cysteine-type endopeptidase inhibitor activity; GO:0005576 extracellular region; GO:0008284 positive regulation of cell population proliferation; GO:0010718 positive regulation of epithelial to mesenchymal transition; GO:0010950 positive regulation of endopeptidase activity; GO:0030335 positive regulation of cell migration; GO:0030414 peptidase inhibitor activity; GO:0031410 cytoplasmic vesicle; GO:0031982 vesicle; GO:0035425 autocrine signaling; GO:0035578 azurophil granule lumen; GO:0038001 paracrine signaling; GO:0043086 negative regulation of catalytic activity; GO:0043312 neutrophil degranulation; GO:0043508 negative regulation of JUN kinase activity; GO:0045861 negative regulation of proteolysis; GO:0046718 viral entry into host cell
SPRR2A	Small Proline Rich Protein 2A	GO:0005829 cytosol; GO:0001533 cornified envelope; GO:0005737 cytoplasm; GO:0005515 protein binding; GO:0008544 epidermis development; GO:0030216 keratinocyte differentiation; GO:0031424 keratinization; GO:0070268 cornification

TCN1	Transcobalamin-1	GO:0005576 extracellular region; GO:0015889 cobalamin transport; GO:0005615 extracellular space; GO:0031419 cobalamin binding; GO:0006811 ion transport; GO:0006824 cobalt ion transport; GO:0009235 cobalamin metabolic process; GO:0035580 specific granule lumen; GO:0043312 neutrophil degranulation; GO:1904724 tertiary granule lumen
c10orf99	Chromosome 10 Open Reading Frame 99	GO:0005576 extracellular region; GO:0001664 G protein-coupled receptor binding; GO:0007186 G protein-coupled receptor signaling pathway; GO:0008009 chemokine activity; GO:0048247 lymphocyte chemotaxis; GO:0051782 negative regulation of cell division; GO:0005125 cytokine activity; GO:0005515 protein binding; GO:0005615 extracellular space; GO:0006935 chemotaxis; GO:0042742 defense response to bacterium; GO:0048018 receptor ligand activity; GO:0050830 defense response to Gram-positive bacterium; GO:0050832 defense response to fungus; GO:1902807 negative regulation of cell cycle G1/S phase transition; GO:2000404 regulation of T cell migration
AKR1B10	Aldo-Keto Reductase Family 1 Member B10	GO:0016491 oxidoreductase activity; GO:0055114 oxidation-reduction process; GO:0001523 retinoid metabolic process; GO:0001758 retinal dehydrogenase activity; GO:0008106 alcohol dehydrogenase (NADP+) activity; GO:0016488 farnesol catabolic process; GO:0042572 retinol metabolic process; GO:0044597 daunorubicin metabolic process; GO:0044598 doxorubicin metabolic process; GO:0045550 geranylgeranyl reductase activity; GO:0047718 indanol dehydrogenase activity; GO:0052650 NADP-retinol dehydrogenase activity; GO:0110095 cellular detoxification of aldehyde
SPRR2B	Small Proline Rich Protein 2B	GO:0005829 cytosol; GO:0001533 cornified envelope; GO:0005737 cytoplasm; GO:0008544 epidermis development; GO:0030216 keratinocyte differentiation; GO:0031424 keratinization; GO:0070268 cornification
KRT16	Keratin 16	GO:0002009 morphogenesis of an epithelium; GO:0005198 structural molecule activity; GO:0005200 structural constituent of cytoskeleton; GO:0005515 protein binding; GO:0005634 nucleus; GO:0005829 cytosol; GO:0005856 cytoskeleton; GO:0005882 intermediate filament; GO:0006954 inflammatory response; GO:0007010 cytoskeleton organization; GO:0007568 aging; GO:0030216 keratinocyte differentiation; GO:0070062 extracellular exosome
CSTA	Cystatin A	GO:0010951 negative regulation of endopeptidase activity; GO:0004869 cysteine-type endopeptidase inhibitor activity; GO:0005737 cytoplasm; GO:0005829 cytosol; GO:0001533 cornified envelope; GO:0002020 protease binding; GO:0004866 endopeptidase inhibitor activity; GO:0005615 extracellular space; GO:0005654 nucleoplasm; GO:0010466 negative regulation of peptidase activity; GO:0030414 peptidase inhibitor activity
LCE3D	Late cornified envelope protein 3D	GO:0005515 protein binding; GO:0005829 cytosol; GO:0008544 epidermis development; GO:0031424 keratinization; GO:0070268 cornification
HEPHL1	Ferroxidase	GO:0004322 ferroxidase activity; GO:0005739 mitochondrion; GO:0055114 oxidation-reduction process; GO:0006783 heme biosynthetic process; GO:0006811 ion transport; GO:0006879 cellular iron ion homeostasis; GO:0008199 ferric iron binding; GO:0016226 iron-sulfur cluster assembly; GO:0016491 oxidoreductase activity; GO:0055072 iron ion homeostasis

KRT6A	Keratin 6A	GO:0001899 negative regulation of cytolysis by symbiont of host cells; GO:0002009 morphogenesis of an epithelium; GO:0005200 structural constituent of cytoskeleton; GO:0005515 protein binding; GO:0005634 nucleus; GO:0005829 cytosol; GO:0005882 intermediate filament; GO:0007010 cytoskeleton organization; GO:0008284 positive regulation of cell proliferation; GO:0016020 membrane; GO:0030154 cell differentiation; GO:0045095 keratin filament
KRT17	Keratin 17	GO:0002009 morphogenesis of an epithelium; GO:0005198 structural molecule activity; GO:0005515 protein binding; GO:0005737 cytoplasm; GO:0005829 cytosol; GO:0005882 intermediate filament; GO:0030307 positive regulation of cell growth; GO:0031069 hair follicle morphogenesis; GO:0031424 keratinization; GO:0045109 intermediate filament organization; GO:0045111 intermediate filament cytoskeleton; GO:0071944 cell periphery
SPRR2D	Small Proline Rich Protein 2D	GO:0001533 cornified envelope; GO:0005737 cytoplasm; GO:0005829 cytosol; GO:0008544 epidermis development; GO:0031424 keratinization; GO:0070268 cornification
SPRR1B	Small Proline Rich Protein 1B	GO:0001533 cornified envelope; GO:0005198 structural molecule activity; GO:0005737 cytoplasm; GO:0005829 cytosol; GO:0008544 epidermis development; GO:0018149 peptide cross-linking; GO:0030216 keratinocyte differentiation; GO:0031424 keratinization; GO:0070268 cornification
FABP5	Fatty Acid Binding Protein 5	GO:0001972 retinoic acid binding; GO:0005324 long-chain fatty acid transporter activity; GO:0005504 fatty acid binding; GO:0005515 protein binding; GO:0005576 extracellular region; GO:0005615 extracellular space; GO:0005634 nucleus; GO:0005654 nucleoplasm; GO:0005737 cytoplasm; GO:0008289 lipid binding
SPRR2F	Small Proline Rich Protein 2F	GO:0001533 cornified envelope; GO:0005737 cytoplasm; GO:0005829 cytosol; GO:0008544 epidermis development; GO:0030216 keratinocyte differentiation; GO:0031424 keratinization; GO:0070268 cornification
S100A8	S100 Calcium Binding Protein A8	GO:0001816 cytokine production; GO:0002224 toll-like receptor signaling pathway; GO:0002376 immune system process; GO:0002523 leukocyte migration involved in inflammatory response; GO:0002526 acute inflammatory response; GO:0005509 calcium ion binding; GO:0005515 protein binding; GO:0005576 extracellular region; GO:0005615 extracellular space; GO:0005623 cell; GO:0005634 nucleus; GO:0005737 cytoplasm; GO:0008017 microtubule binding; GO:0008270 zinc ion binding; GO:0035662 Toll-like receptor 4 binding;
IFI27	Interferon Alpha Inducible Protein 27	GO:0000122 negative regulation of transcription by RNA polymerase II; GO:0001102 RNA polymerase II activating transcription factor binding; GO:0002376 immune system proces; GO:0003674 molecular_function; GO:0005515 protein binding; GO:0005521 lamin binding; GO:0005634 nucleus; GO:0005635 nuclear envelope; GO:0005637 nuclear inner membrane; GO:0005739 mitochondrion; GO:0005741 mitochondrial outer membrane; GO:0006915 apoptotic process; GO:0016032 viral process; GO:0042802 identical protein binding; GO:0043161 proteasome-mediated ubiquitin-dependent protein catabolic process
TMPRSS11D	Transmembrane Serine Protease 11D	GO:0004252 serine-type endopeptidase activity; GO:0005576 extracellular region; GO:0005886 plasma membrane; GO:0005887 integral component of plasma membrane; GO:0006508 proteolysis; GO:0007585 respiratory gaseous exchange; GO:0008233 peptidase activity; GO:0008236 serine-type peptidase activity; GO:0016020 membrane; GO:0016021 integral component of membrane; GO:0016787 hydrolase activity

KYNU	Kynureninase	GO:0003824 catalytic activity; GO:0005654 nucleoplasm; GO:0005737 cytoplasm; GO:0005739 mitochondrion; GO:0005829 cytosol; GO:0006569 tryptophan catabolic process; GO:0007568 aging; GO:0009435 NAD biosynthetic process; GO:0016787 hydrolase activity; GO:0019363 pyridine nucleotide biosynthetic process; GO:0030170 pyridoxal phosphate binding; GO:0019441 tryptophan catabolic process to kynurenine; GO:0030429 kynureninase activity; GO:0042803 protein homodimerization activity;
S100A12	S100 Calcium Binding Protein A12	GO:0002376 immune system process; GO:0002548 monocyte chemotaxis; GO:0005507 copper ion binding; GO:0005509 calcium ion binding; GO:0005515 protein binding; GO:0005576 extracellular region; GO:0005634 nucleus; GO:0005737 cytoplasm; GO:0005829 cytosol; GO:0005856 cytoskeleton; GO:0006805 xenobiotic metabolic process; GO:0006954 inflammatory response; GO:0008270 zinc ion binding; GO:0030593 neutrophil chemotaxis; GO:0046872 metal ion binding
CXCL8	C-X-C Motif Chemokine Ligand 8	GO:0001525 angiogenesis; GO:0002237 response to molecule of bacterial origin; GO:0005125 cytokine activity; GO:0005153 interleukin-8 receptor binding; GO:0005515 protein binding; GO:0005576 extracellular region; GO:0005615 extracellular space; GO:0006935 chemotaxis; GO:0006952 defense response; GO:0006954 inflammatory response; GO:0008009 chemokine activity; GO:0045236 CXCR chemokine receptor binding

Supplementary Table 2. Biological functions of the proteins encoded by the upregulated DEGs in the study by Kulski et al.

Gene Name	Protein Name	Functions
JUNB	JunB Proto-Oncogene	GO:0000122 negative regulation of transcription by RNA polymerase II; GO:0000785 chromatin; GO:0000790 nuclear chromatin; GO:0000977 RNA polymerase II regulatory region sequence-specific DNA binding; GO:0000978 RNA polymerase II proximal promoter sequence-specific DNA binding; GO:0000981 DNA-binding transcription factor activity, RNA polymerase II-specific; GO:0001228 DNA-binding transcription activator activity, RNA polymerase II-specific; GO:0001570 vasculogenesis; GO:0001649 osteoblast differentiation; GO:0001701 in utero embryonic development; GO:0001829 trophectodermal cell differentiation; GO:0003677 DNA binding; GO:0005634 nucleus; GO:0005654 nucleoplasm; GO:0005667 transcription factor complex
YWHAB	Tyrosine 3-Monooxygenase/Tryptophan 5-Monooxygenase Activation Protein Beta	GO:0000165 MAPK cascade; GO:0005515 protein binding; GO:0005634 nucleus; GO:0005737 cytoplasm; GO:0005739 mitochondrion; GO:0005773 vacuole; GO:0005774 vacuolar membrane; GO:0006605 protein targeting; GO:0008022 protein C-terminus binding; GO:0016032 viral process; GO:0019899 enzyme binding; GO:0019904 protein domain specific binding; GO:0035308 negative regulation of protein dephosphorylation; GO:0035329 hippo signaling; GO:0042802 identical protein binding
LAMP3	Lysosomal Associated Membrane Protein 3	GO:0002250 adaptive immune response; GO:0002376 immune system process; GO:0005764 lysosome; GO:0005765 lysosomal membrane; GO:0005769 early endosome; GO:0005886 plasma membrane; GO:0010506 regulation of autophagy; GO:0010628 positive regulation of gene expression; GO:0016020 membrane; GO:0035455 response to interferon-alpha
SEC61G	SEC61 Translocon Subunit Gamma	GO:0005515 protein binding; GO:0005783 endoplasmic reticulum; GO:0005789 endoplasmic reticulum membrane; GO:0005829 cytosol; GO:0006605 protein targeting; GO:0006886 intracellular protein transport; GO:0008320 protein transmembrane transporter activity; GO:0015031 protein transport; GO:0015450 P-P-bond-hydrolysis-driven protein transmembrane transporter activity; GO:0016020 membrane; GO:0016021 integral component of membrane; GO:0031204 posttranslational protein targeting to membrane, translocation; GO:0045047 protein targeting to ER
KIAA0101	PCNA Clamp Associated Factor	GO:0003682 chromatin binding; GO:0005515 protein binding; GO:0005634 nucleus; GO:0005654 nucleoplasm; GO:0005737 cytoplasm; GO:0005813 centrosome; GO:0006260 DNA replication; GO:0006281 DNA repair; GO:0006974 cellular response to DNA damage stimulus; GO:0007098 centrosome cycle; GO:0009411 response to UV; GO:0048471 perinuclear region of cytoplasm
CSTA	Cystatin A	GO:0010951 negative regulation of endopeptidase activity; GO:0004869 cysteine-type endopeptidase inhibitor activity; GO:0005737 cytoplasm; GO:0005829 cytosol; GO:0001533 cornified envelope; GO:0002020 protease binding; GO:0004866 endopeptidase inhibitor activity; GO:0005615 extracellular space; GO:0005654 nucleoplasm; GO:0010466 negative regulation of peptidase activity; GO:0030414 peptidase inhibitor activity

OAS1	2'-5'-Oligoadenylate Synthetase 1	GO:0000166 nucleotide binding; GO:0001730 2'-5'-oligoadenylate synthetase activity; GO:0003723 RNA binding; GO:0003725 double-stranded RNA binding; GO:0005515 protein binding; GO:0005576 extracellular region; GO:0005634 nucleus; GO:0005654 nucleoplasm; GO:0005737 colocalizes with cytoplasm; GO:0005739 mitochondrion; GO:0006006 glucose metabolic process; GO:0006955 immune response; GO:0009615 response to virus; GO:0042593 glucose homeostasis
CCL20	C-C Motif Chemokine Ligand 20	GO:0002548 monocyte chemotaxis; GO:0005125 cytokine activity; GO:0005515 protein binding; GO:0005576 extracellular region; GO:0005615 extracellular space; GO:0006935 chemotaxis; GO:0006954 inflammatory response; GO:0006955 immune response; GO:0007165 signal transduction; GO:0008009 chemokine activity; GO:0031731 CCR6 chemokine receptor binding; GO:0048020 CCR chemokine receptor binding
TGM1	Transglutaminase 1	GO:0001533 cornified envelope; GO:0003810 protein-glutamine gamma-glutamyltransferase activity; GO:0005515 protein binding; GO:0005829 cytosol; GO:0005886 plasma membrane; GO:0006464 cellular protein modification process; GO:0010838 positive regulation of keratinocyte proliferation; GO:0016020 membrane; GO:0016740 transferase activity; GO:0016746 transferase activity, transferring acyl groups; GO:0018149 peptide cross-linking; GO:0030216 keratinocyte differentiation; GO:0031424 keratinization; GO:0031224 intrinsic component of membrane; GO:0046872 metal ion binding
SEC61B	SEC61 Translocon Subunit Beta	GO:0003723 RNA binding; GO:0005086 ARF guanyl-nucleotide exchange factor activity; GO:0005515 protein binding; GO:0005783 endoplasmic reticulum; GO:0005784 Sec61 translocon complex; GO:0005789 endoplasmic reticulum membrane; GO:0005829 cytosol; GO:0006616 SRP-dependent cotranslational protein targeting to membrane, translocation; GO:0006886 intracellular protein transport; GO:0016020 membrane; GO:0015031 protein transport; GO:0030433 ubiquitin-dependent ERAD pathway; GO:0030970 retrograde protein transport, ER to cytosol; GO:0048408 epidermal growth factor binding
GBA	Glucosylceramidase Beta	GO:0004348 glucosylceramidase activity; GO:0005102 signaling receptor binding; GO:0005124 scavenger receptor binding; GO:0005515 protein binding; GO:0005615 extracellular space; GO:0005764 lysosome; GO:0005765 lysosomal membrane; GO:0005783 endoplasmic reticulum; GO:0005794 Golgi apparatus; GO:0006629 lipid metabolic process; GO:0006665 sphingolipid metabolic process; GO:0006680 glucosylceramide catabolic process; GO:0006687 glycosphingolipid metabolic process; GO:0006914 autophagy; GO:0016740 transferase activity
H2AFY	H2A Histone Family Member Y	GO:0000122 negative regulation of transcription by RNA polymerase II; GO:0000182 rDNA binding; GO:0000228 nuclear chromosome; GO:0000784 nuclear chromosome, telomeric region; GO:0000785 chromatin GO:0000786 nucleosome; GO:0000790 nuclear chromatin; GO:0000976 transcription regulatory region sequence-specific DNA binding; GO:0000977 RNA polymerase II regulatory region sequence-specific DNA binding; GO:0000979 RNA polymerase II core promoter sequence-specific DNA binding; GO:0003677 DNA binding; GO:0006325 chromatin organization; GO:0006334 nucleosome assembly; GO:0006342 chromatin silencing; GO:0007549 dosage compensation

UBE2L6	Ubiquitin Conjugating Enzyme E2 L6	GO:0000151 ubiquitin ligase complex; GO:0000166 nucleotide binding; GO:0000209 protein polyubiquitination; GO:0004842 ubiquitin-protein transferase activity; GO:0005515 protein binding; GO:0005524 ATP binding; GO:0005634 nucleus; GO:0005654 nucleoplasm; GO:0005829 cytosol; GO:0006464 cellular protein modification process; GO:0006511 ubiquitin-dependent protein catabolic process; GO:0016567 protein ubiquitination; GO:0016740 transferase activity; GO:0019941 modification-dependent protein catabolic process
GM2A	GM2 Ganglioside Activator	GO:0001573 ganglioside metabolic process; GO:0004563 beta-N-acetylhexosaminidase activity; GO:0005319 lipid transporter activity; GO:0005576 extracellular region; GO:0005737 cytoplasm; GO:0005764 lysosome; GO:0006629; lipid metabolic process; GO:0006665 sphingolipid metabolic process; GO:0006687 glycosphingolipid metabolic process; GO:0006689 ganglioside catabolic process; GO:0008047 enzyme activator activity; GO:0009898 cytoplasmic side of plasma membrane; GO:0016004 phospholipase activator activity; GO:0016323 basolateral plasma membrane; GO:0016787 hydrolase activity
SULT2B1	Sulfotransferase Family 2B Member 1	GO:0003676 nucleic acid binding; GO:0004027 alcohol sulfotransferase activity; GO:0005515 protein binding; GO:0005634 nucleus; GO:0005737 cytoplasm; GO:0005783 endoplasmic reticulum; GO:0005829 cytosol; GO:0006629 lipid metabolic process; GO:0008202 steroid metabolic process; GO:0008203 cholesterol metabolic process; GO:0008285 negative regulation of cell proliferation; GO:0008146 sulfotransferase activity; GO:0015485 cholesterol binding; GO:0043231 intracellular membrane-bounded organelle
P4HB	Prolyl 4-Hydroxylase Subunit Beta	GO:0003723 RNA binding; GO:0003756 protein disulfide isomerase activity; GO:0003779 actin binding; GO:0004656 contributes to procollagen-proline 4-dioxygenase activity; GO:0005178 integrin binding; GO:0005576 extracellular region; GO:0005623 cell; GO:0005783 endoplasmic reticulum; GO:0005788 endoplasmic reticulum lumen; GO:0005793 endoplasmic reticulum-Golgi intermediate compartment; GO:0006457 protein folding; GO:0018401 peptidyl-proline hydroxylation to 4-hydroxy-L-proline; GO:0034378 chylomicron assembly; GO:0034379 very-low-density lipoprotein particle assembly; GO:0034976 response to endoplasmic reticulum stress
RER1	Retention In Endoplasmic Reticulum Sorting Receptor 1	GO:0000139 Golgi membrane; GO:0003674 molecular function; GO:0005783 endoplasmic reticulum; GO:0005793 endoplasmic reticulum-Golgi intermediate compartment; GO:0005794 Golgi apparatus; GO:0005886 plasma membrane; GO:0006621 protein retention in ER lumen; GO:0006890 retrograde vesicle-mediated transport, Golgi to ER; GO:0007528 neuromuscular junction development; GO:0033130 acetylcholine receptor binding; GO:0071340 skeletal muscle acetylcholine-gated channel clustering; GO:1903078 positive regulation of protein localization to plasma membrane
PSMB6	Proteasome 20S Subunit Beta 6	GO:0000165 MAPK cascade; GO:0000209 protein polyubiquitination; GO:0000502 proteasome complex; GO:0002223 stimulatory C-type lectin receptor signaling pathway; GO:0002479 antigen processing and presentation of exogenous peptide antigen via MHC class I, TAP-dependent; GO:0004175 endopeptidase activity; GO:0004298 threonine-type endopeptidase activity; GO:0005515 protein binding; GO:0005634 nucleus; GO:0005654 nucleoplasm; GO:0005737 cytoplasm; GO:0005829 cytosol; GO:0006508 proteolysis; GO:0008233 peptidase activity; GO:0016787 hydrolase activity

NMI	N-Myc And STAT Interactor	GO:0003712 transcription coregulator activity; GO:0005515 protein binding; GO:0005654 nucleoplasm; GO:0005737 cytoplasm; GO:0005829 cytosol; GO:0006366 transcription by RNA polymerase II; GO:0006954 inflammatory response; GO:0007259 JAK-STAT cascade; GO:0032480 negative regulation of type I interferon production; GO:0042802 identical protein binding; GO:0045355 negative regulation of interferon-alpha biosynthetic process
IVL	Involucrin	GO:0001533 cornified envelope; GO:0005515 protein binding; GO:0005737 cytoplasm; GO:0005813 centrosome; GO:0005829 cytosol; GO:0010224 response to UV-B; GO:0016604 nuclear body; GO:0018153 isopeptide cross-linking via N6-(L-isoglutamyl)-L-lysine; GO:0030216 keratinocyte differentiation; GO:0031424 keratinization

Supplementary Table 3. DEGs from pathways related to lipid metabolism

Lipid processes	DEGs	Full name	Expression	Location	References
Biosynthesis of unsaturated fatty acids / Fatty acid elongation	*ACOT2*	Acyl-CoA thioesterase 2	Downregulated	14q24.3	Malik et al. [1]
Fatty acid beta-oxidation (peroxisome)	*ACOX2*	Acyl-CoA oxidase 2	Downregulated	3p14.3	Malik et al. [1]
Retinoid metabolism and transport / Glycerolipid metabolism	*AKR1B10*	Aldo-keto reductase family 1 member B10	Upregulated	7q33	Gao et al. (93)<br>Gudjonsson et al. (68)<br>Li et al. (72)<br>Ahn et al. (76)<br>Pasquali et al. (81)
Metabolism of lipids and lipoproteins / metabolism of steroid hormones	*AKR1B15*	Aldo-keto reductase family 1 member B15	Downregulated	7q33	Gao et al. (93)
Fatty acid metabolism/ arachidonic acid metabolism	*ALOX12B*	Arachidonate 12-lipoxygenase	Upregulated	17p13.1	Zolotarenko et al. [2]
arachidonic acid metabolism / Wax biosynthesis	*AWAT1*	Acyl-CoA was alcohol acyltransferase 1	Downregulated	Xq13.1	Malik et al. [1]<br>Zolotarenko et al. [3]<br>Li et al. (72)
Retinol metabolism / Glycerophospholipid biosynthesis	*AWAT2*	Acyl-CoA wax alcohol acyltransferase	Downregulated	Xq13.1	Zolotarenko et al. [2]<br>Li et al. (72)
Metabolism of lipids and lipoproteins	*CIDEC*	Cell death inducing DFFA like effector C	Downregulated	3p25.3	Malik et al. [1]
Fatty acid beta oxidation / Peroxisomal lipid metabolism	*CRAT*	Carnitine acetyltransferase	Downregulated	9q34.1	Zolotarenko et al. [2]
Acyl-chain remodeling / Glycerophospholipid biosynthesis / Phospholipid metabolism	*DGAT2L6*	Diacylglycerol O-acyltransferase 2-like 6	Downregulated	Xq13.1	Zolotarenko et al. [2]<br>Li et al. (72)
Biosynthesis of unsaturated fatty acids / Fatty acid elongation / alpha-linolenic and linoleic acid metabolism	*ELOVL3*	Fatty acid elongase 3	Downregulated	10q24.32	Malik et al. [1]<br>Zolotarenko et al. [2]<br>Gudjonsson et al. (67)<br>Gudjonsson et al. (68)<br>Li et al. (72)
Metabolism of lipids and lipoproteins / Regulation of lipolysis in adipocytes / Lipid digestion, mobilization, and transport	*FABP4*	Fatty acid binding protein 4	Downregulated	8q21.13	Malik et al. [1]<br>Zolotarenko et al. [2]

Metabolism of lipids and lipoproteins / Lipid digestion, mobilization, and transport / PPAR signaling pathway	*FABP5*	Fatty acid binding protein 5	Upregulated	8q21.13	Bowcock et al. (60)<br>Mee et al. (64)
Metabolism of lipids and lipoproteins / Lipid digestion, mobilization, and transport / PPAR signaling pathway	*FABP7*	Fatty acid binding protein 7	Downregulated	6q22.31	Malik et al. [1]<br>Gudjonsson et al. (68)
Alpha linolenic and linoleic acid metabolism / Biosynthesis of unsaturated fatty acids / Regulation of lipid metabolism by PPARalpha	*FADS1*	Fatty acid desaturase 1	Downregulated	11q12.2	Malik et al. [1]<br>Gudjonsson et al. (68)
Alpha linolenic and linoleic acid metabolism / Biosynthesis of unsaturated fatty acids / Fatty acid beta-oxidation (peroxisome)	*FADS2*	Fatty acid desaturase 2	Downregulated	11q12-q13.1	Malik et al. [1]<br>Zolotarenko et al. [2]
Metabolism of lipids and lipoproteins / Peroxisome / Wax biosynthesis	*FAR2*	Fatty acyl-CoA reductase 2	Downregulated	12p11.22	Malik et al. [1]
Metabolism of lipids and lipoproteins / Sphingolipid de novo biosynthesis / Fatty acid alpha-oxidation III	*FA2H*	Fatty acid 2-Hydroxylase	Downregulated	16q23.1	Malik et al. [1]
Sphingolipid metabolism / Lysosome / Metabolism of lipids and lipoproteins	*GBA*	Glucosylceramidase Beta	Upregulated	1q22	Kulski et al. (63)
Sphingolipid metabolism / Lysosome / metabolism of lipids and lipoproteins	*GM2A*	GM2 Ganglioside Activator	Upregulated	5q33.1	Kulski et al. (63)
Peroxisomal lipid metabolism / Metabolism of lipids and lipoproteins	*HAO2*	Hydroxyacid oxidase 2 (long chain)	Downregulated	1p13.3-p13.1 Cluster C1 (PSORS7)	Malik et al. [1]<br>Zolotarenko et al. [2]<br>Li et al. (72)
Superpathway of cholesterol biosynthesis / Regulation of lipid metabolism by PPARalpha / Terpenoid backbone biosynthesis	*HMGCS2*	3-Hydroxy-3-Methylglutaryl-CoA Synthase 2	Downregulated	1p12	Malik et al. [1]
Steroid biosynthesis / Metabolism of steroid hormones / Cortisol synthesis and secretion	*HSD3B1*	Hydroxyl-dela-5-steroid dehydrogenase, 3 beta- and steroid delta-isomerase 1	Downregulated	1p13.1 Cluster C1 (PSORS7)	Zolotarenko et al. [2]<br>Gudjonsson et al. (68)
No pathway	*HSD3BP2*	Hydroxyl-delta-5-steroid dehydrogenase, 3 beta, pseudogene 2	Downregulated	1p13.1 Cluster C1 (PSORS7)	Zolotarenko et al. [2]
Alpha linolenic, linoleic and arachidonic acid metabolism / Phospholipase D signaling pathway	*JMJD7-PLA2G4B*	Jumonji Domain Containing 7-phospholipase A2, Group IVB (cytosolic) Read-Through	Upregulated	15q15.1	Gao et al. (93)
Cholesterol metabolism / Glycerolipid metabolism / PPAR signaling pathway	*LPL*	Lipoprotein lipase	Downregulated	8p21.3	Malik et al. [1]

Triglyceride Biosynthesis / Glycerolipid metabolism / Regulation of lipid metabolism by PPARalpha	*MOGAT1*	Monoacylglycero O-Acyltransferase 1	Downregulated	2q36.1	Malik et al. [1]
Triglyceride synthesis / Glycerolipid metabolism / Regulation of lipid metabolism by PPARalpha	*MOGAT2*	Monoacylglycerol O-acyltransferase 2	Downregulated	11q13.5 Cluster C3	Zolotarenko et al. [2]
Lipoprotein metabolism / metabolism of lipids and lipoproteins	*P4HB*	Prolyl 4-Hydroxylase Subunit Beta	Upregulated	17q25.3	Kulski et al. (63)
Phospholipid metabolism / alpha-linolenic, linoleic and arachidonic acid metabolism / Eicosanoid synthesis	*PLA2G2A*	Phospholipase A2 Group IIA	Upregulated	1p36.13	Gao et al. (93)
Phospholipid metabolism / alpha-linolenic, linoleic and arachidonic acid metabolism / Fat digestion and absorption	*PLA2G2F*	Phospholipase A2 Group IIF	Upregulated	1p36.12	Gao et al. (93)
Phospholipid metabolism / alpha-linolenic, linoleic and arachidonic acid metabolism / Fat digestion and absorption	*PLA2G3*	Phospholipase A2 Group III	Upregulated	22q12.2	Gao et al. (93)
Phospholipid metabolism / alpha-linolenic, linoleic and arachidonic acid metabolism	*PLA2G4B*	Phospholipase A2 Group IVB	Upregulated	15q15.1	Gao et al. (93)
Phospholipid metabolism / alpha-linolenic, linoleic and arachidonic acid metabolism / Phospholipase D signaling pathway	*PLA2G4D*	Phospholipase A2 Group IVD	Upregulated	15q15.1	Gao et al. (93)
Phospholipid metabolism / alpha-linolenic, linoleic and arachidonic acid metabolism / Phospholipase D signaling pathway	*PLA2G4E*	Phospholipase A2 Group IVE	Upregulated	15q15.1	Gao et al. (93)
Development angiotensin activation of ERK / Aldosterone synthesis and secretion	*PLCB4*	Phospholipase C Beta 4	Downregulated	20qp12.3	Malik et al. [1]
Phospholipid metabolism / Adipogenesis / Eicosanoid Synthesis	*PNPLA3*	Patatin Like Phospholipase Domain containing 3	Downregulated	22q13.31	Malik et al. [1]
Metabolism of lipids and lipoproteins / Regulation of lipid metabolism by PPARalpha	*PNPLA5*	Patatin-like phospholipase domain containing 5	Downregulated	22q13.31	Zolotarenko et al. [2] Li et al. (72)
Gene expression / Metabolism of lipids and lipoproteins / Regulation of lipid metabolism by PPARalpha	*PPARG*	Peroxisome Proliferator Activated Receptor Gamma	Downregulated	3p25.2	Malik et al. [1] Zolotarenko et al. [2]
Cholesterol metabolism / Lipoprotein metabolism / Steroid biosynthesis	*SOAT1*	Sterol O-Acyltransferase 1	Downregulated	1q25.2	Malik et al. [1]
Steroid hormone biosynthesis	*SULT2B1*	Sulfotransferase Family 2B Member 1	Upregulated	19q13.33	Kulski et al. (63)

Supplementary references

1. Malik, K.; He, H.; Huynh, T.N.; Tran, G.; Mueller, K.; Doytcheva, K.; Renert-Yuval, Y.; Czarnowicki, T.; Magidi, S.; Chou, M., et al. Ichthyosis molecular fingerprinting shows profound T(H)17 skewing and a unique barrier genomic signature. *The Journal of allergy and clinical immunology* **2019**, *143*, 604-618, doi:10.1016/j.jaci.2018.03.021.
2. Zolotarenko, A.; Chekalin, E.; Mesentsev, A.; Kiseleva, L.; Gribanova, E.; Mehta, R.; Baranova, A.; Tatarinova, T.V.; Piruzian, E.S.; Bruskin, S. Integrated computational approach to the analysis of RNA-seq data reveals new transcriptional regulators of psoriasis. *Experimental & molecular medicine* **2016**, *48*, e268, doi:10.1038/emm.2016.97.
3. Zolotarenko, A.; Chekalin, E.; Mehta, R.; Baranova, A.; Tatarinova, T.V.; Bruskin, S. Identification of Transcriptional Regulators of Psoriasis from RNA-Seq Experiments. *Methods in molecular biology (Clifton, N.J.)* **2017**, *1613*, 355-370, doi:10.1007/978-1-4939-7027-8_14.
